# A Narrative Review of the Role of S-Glutathionylation in Bacteria

**DOI:** 10.3390/microorganisms13030527

**Published:** 2025-02-27

**Authors:** Luca Federici, Michele Masulli, Vincenzo De Laurenzi, Nerino Allocati

**Affiliations:** 1 Department of Innovative Technologies in Medicine and Dentistry, University “G. d’ Annunzio”, 66100 Chieti, Italy; luca.federici@unich.it (L.F.); michele.masulli@unich.it (M.M.); vincenzo.delaurenzi@unich.it (V.D.L.); 2 CAST (Center for Advanced Studies and Technology), University “G. d’ Annunzio”, 66100 Chieti, Italy

**Keywords:** glutathione, GSH, S-glutathionylation, GS-ylation, oxidative stress, post-translational modification

## Abstract

Protein glutathionylation is defined as a reversible, ubiquitous post-translational modification, resulting in the formation of mixed disulfides between glutathione and proteins’ cysteine residues. Glutathionylation has been implicated in several cellular mechanisms ranging from protection from oxidative stress to the control of cellular homeostasis and the cell cycle. A significant body of research has examined the multifaceted effects of this post-translational modification under physiological conditions in eukaryotes, with a particular focus on its impact on the development of various diseases in humans. In contrast, the role of glutathionylation in prokaryotic organisms remains to be extensively investigated. However, there has been a recent increase in the number of studies investigating this issue, providing details about the role of glutathione and other related thiols as post-translational modifiers of selected bacterial proteins. It can be concluded that in addition to the classical role of such thiols in protecting against cysteine oxidation and consequent protein inactivation, many more specialized roles of glutathionylation in bacterial pathogenicity, virulence, interspecies competition and survival, and control of gene expression are emerging, and new ones may emerge in the future. In this short review, we aim to summarize the current state-of-the-art in this field of research.

## 1. Introduction

S-Glutathionylation (GS-ylation) is an important and ubiquitous reversible post-translational modification (PTM) that results in the formation of mixed disulfides between glutathione (GSH) and selected cysteine residues of proteins [1]. It is recognized as one of the most important post-translational mechanisms and regulates various cellular processes under physiological conditions, such as protection against oxidative stress [2]. In eukaryotes, under pathological conditions, deregulated GS-ylation is associated with cardiovascular, pulmonary, and neurodegenerative diseases as well as cancer and diabetes [2].

**Figure 1:**
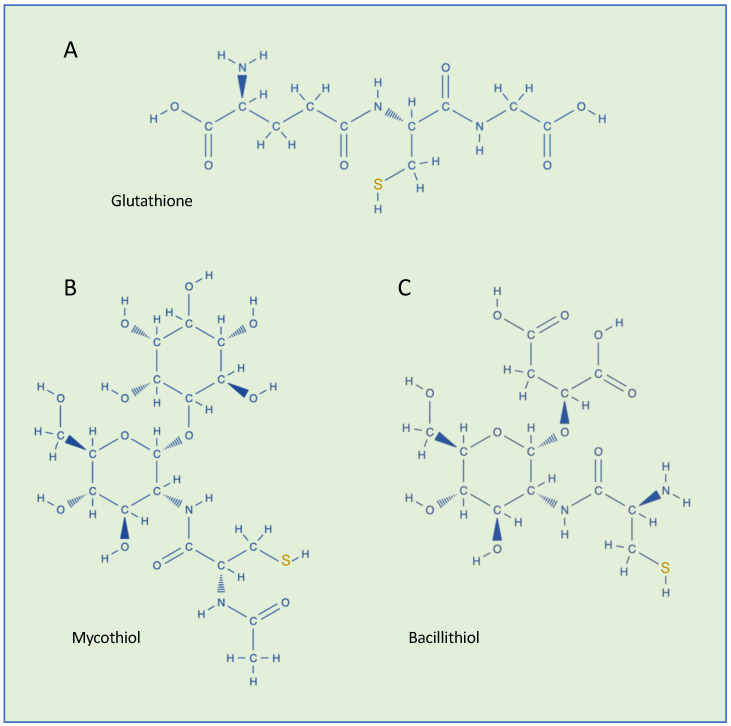
Chemical structures of low-molecular-weight thiols found in bacteria. (**A**) Glutathione (GSH, γ-Glu-Cys-Gly), (**B**) mycothiol (MSH, AcCys-GlcN-Ins), and (**C**) bacillithiol (BSH, Cys-GlcN-Mal).

GSH (L-γ-glutamyl-L-cysteinyl-glycine) is the most abundant eukaryotic and prokaryotic low-molecular-weight thiol, with cellular concentrations in the millimolar range, and is a major regulator of redox status and redox signaling in cells (Figure 1A) [3,4].

GS-ylation has been studied mainly in eukaryotes [2,5,6], including a number of studies focused on unicellular eukaryotic microorganisms such as protozoa, microalgae, and yeasts [7,8,9,10,11,12,13].

Conversely, to date, little is known about GS-ylation in bacteria. In prokaryotes, GSH is widely distributed in Gram-negative aerobic bacteria and in a few Gram-positive strains [14,15]. Like eukaryotes, in addition to its role in the defense of the cell against oxidative stress, GSH participates in many other cellular reactions [14,15]. Some bacterial Gram-positive species that lack the genes and the enzymes necessary to synthesize GSH can acquire thiol from the extracellular environment [16]. In other bacterial species, proteins are post-translationally modified using different thiols. Gram-positive *Actinomycetes* and *Firmicutes* use mycothiol and bacillithiol, respectively, as thiol-redox buffers to maintain the reduced state of the cytoplasm (Figure 1B,C) [17]. Under oxidative stress, protein S-bacillithiolation and S-mycothiolation have been observed in these bacteria [17]. Thiols are also found in archaea [18] but, currently, S-thiolation has not been reported in these microorganisms. However, since all aerobic organisms are subject to oxidative stress, S-thiolation is expected to occur also in archaea [18].

In this paper, we attempt to provide a narrative review of the state of the art in bacterial GS-ylation research, with a particular focus on recent studies highlighting the involvement of protein GS-ylation in cellular mechanisms.

## 2. Methods

Although this work is a “narrative review” and therefore aims to be mainly descriptive, not involving a systematic search of the literature, the cited articles were retrieved following the guidelines for systematic reviews. We searched articles published up to 13 January 2025 in the PubMed and Web of Science databases, the Google Scholar search engine, and the social network ResearchGate. There were no restrictions on the language or region. We used several keywords for retrieving cited articles, such as: “S-glutathionylation” and “microorganisms or bacteria”. We have retrieved the complete list of abstracts, and the papers that followed the defined criteria for this study were reviewed in full. Additional articles were manually searched by checking the reference lists of already included papers. Two authors (N.A. and L.F.) independently examined all the search results.

## 3. GS-ylation in Bacteria

### 3.1. Streptococcus

The Gram-positive bacterium *Streptococcus* is a genus that encompasses a vast array of species, the majority of which are either commensal or pathogenic for humans and animals. In contrast to *S. agalactiae*, which employs a bifunctional enzyme encoded by a single gene to synthesize GSH [19], pathogenic streptococci, including *S. mutans* and *S. pneumoniae*, lack the genes required for its biosynthesis [20,21]. Consequently, they import GSH from the surrounding environment [16].

In a recent report, the role of GS-ylation in *S. mutants*, considered to be the major pathogen in the initiation of human dental caries, has been investigated, with a focus on its effect on the interspecies competition within the oral streptococcal biofilm [22]. GS-ylation of a thioredoxin-like protein, at Cys41, protects the bacterium from the toxic effects of ROS derived from hostile bacteria, such as *S. sanguinis* and *S. gordonii*, which are pioneer colonizers of the dental plaque that produces high amounts of hydrogen peroxide during aerobic metabolism as a competitive strategy [22].

*S. pneumoniae* is a facultative anaerobic microorganism that is responsible for the onset of acute bacterial infections [23]. The bacterium is an important agent of community-acquired pneumonia infections in humans, and it is responsible for millions of deaths worldwide. During infections, *S. pneumoniae* is phagocytosed by host immune cells, which produce ROS as well as the highly reactive oxidant hypochlorous acid within the respiratory burst, thereby killing the microorganisms. The NmlR regulator (*Neisseria* MeR-like regulator) was identified as being strongly up-regulated in response to hypochlorous acid (HClO) stress in the transcriptome of *S. pneumoniae* D39 [24]. The NmlR regulator is a transcriptional activator that controls the expression of the *nmlR-adhC* operon under various stresses in *S. pneumoniae* [25]. NmlR is related to a large and diverse group of MerR-like regulators [26]. The *adhC* gene encodes a GSH-dependent alcohol dehydrogenase (AdhC). It has been observed that NmlR is involved in the defense against oxidative stress in *S. pneumoniae* [24]. In the repressor state, the Cys52 residues of the two NmlR subunits are reduced. Indeed, the basic structure of MerR proteins is a homodimer [27]. During HClO stress, NmlR is either oxidized to form intersubunit disulfides or GS-ylated, resulting in an increased level of AdhC compared to those constitutively produced. Furthermore, the levels of AdhC were found to be low in the *S. pneumoniae* strain with the NmlR C52A mutation in the presence of HClO. This finding confirms that the conserved Cys52 residue is required for redox-sensing and transcriptional activation by NmlR. Therefore, it was concluded that NmlR functions as a redox-sensing transcriptional activator of the *adhC* gene under HClO stress, depending on the Cys52 status [24].

### 3.2. Escherichia coli

Heat shock proteins (Hsps) form a ubiquitous and conserved protein family across prokaryotic and eukaryotic organisms. They play a crucial role in maintaining cellular protein homeostasis and protecting cells from various forms of stress [28]. Hsps function as molecular chaperones within cells, and they also play a pivotal role in regulating cell signaling, cell cycle, and apoptosis [28]. DnaK is the major bacterial Hsp70 [29], and its GS-ylation has been observed in *E. coli* [30]. It was shown that DnaK becomes inactive under conditions where oxidative stress is accompanied by heat shock and that GS-ylation is one of the factors that contribute to this inactivation. DnaK remains inactive until reducing conditions are restored. Mechanistically, GS-ylation affects the interaction of DnaK with its key partners: DnaJ, GrpE, and σ^32^. The GS-ylation of DnaK has been observed to result in reversible alterations to the secondary structure and tertiary conformation, which in turn leads to a reduction in the binding ability of the protein. The *E. coli* DnaK contains a cysteine residue at position 15, which is highly conserved across different species. It has been noted that this residue undergoes GS-ylation in response to oxidative stress [30].

### 3.3. Yersinia pestis

*Y. pestis* is an extremely virulent pathogen that is responsible for the potentially fatal systemic disease known as plague. To avoid phagocytosis, the bacterium is able to suppress the immune response of white blood cells such as macrophages [31]. The V-antigen (LcrV, Low-Calcium Response V protein) is involved in this process. LcrV is a secreted protein that caps the type III secretion machinery. It has been observed that the GS-ylation of LcrV enhances the pathogenicity of *Y. pestis* in animal models [32]. This was confirmed by the observation that the mutation Cys273Ala improved the animals’ survival [32]. Thus, *Y. pestis* uses GSH in host tissues to activate a virulence strategy that accelerates disease pathogenesis.

### 3.4. Salmonella Typhimurium

*Salmonella enterica* serovar Typhimurium is a Gram-negative primary enteric pathogen that infects both humans and animals [33]. In order to gain a better understanding of the cellular processes underlying infection, the complete proteome of *S*. Typhimurium was investigated by means of a top-down proteomic approach [34]. Top-down mass spectrometry-based proteomics is able to identify and quantify unique proteoforms by analyzing intact proteins [35], providing a wealth of information on protein isoform switching and changes in PTMs. In the case of *S.* Typhimurium, top-down proteomics revealed a differential use of protein S-thiolation. In fact, under basal conditions, the bacterium preferentially used GS-ylation, whereas under infectious-like conditions, *S.* Typhimurium exploited S-cysteinylation. The authors of this study hypothesize that this switch may happen because it is energetically more favorable to synthesize the simpler cysteine moiety, or perhaps because it provides a faster response to changing environmental conditions [34].

### 3.5. Acidithiobacillus caldus

*A. caldus* is a Gram-negative, moderately thermophilic, obligately chemolithotrophic microorganism widely used in bio-leaching processes. It has the capability of oxidizing elemental sulfur and a wide range of reduced inorganic sulfur compounds. It uses energy and electrons derived from sulfur oxidation for carbon dioxide fixation and other anabolic processes. Persulfide dioxygenases catalyze the oxidation of glutathione persulfide (GSSH) and higher homologs with sulfite and GSH as products [36]. In the *A. caldus* strain, persulfide dioxygenase has two surface-exposed cysteines—Cys 87 and Cys 224—involved in catalysis and/or protein stabilization. The GS-ylation of these two cysteines has been reported, suggesting that disulfide formation serves as a protective mechanism against uncontrolled thiol oxidation and the associated loss of enzyme activity [37].

### 3.6. Listeria monocytogenes

Cholesterol-dependent cytolysins (CDCs) consist of a family of oligomeric pore-forming toxins secreted by a range of pathogenic Gram-positive bacteria as virulence factors responsible for disrupting cellular membranes [38]. CDC listeriolysin O is produced by the facultative intracellular *L. monocytogenes* and plays a key role in the rapid bacterial escape from the phagolysosome into the cytoplasm where the bacterium reproduces [38]. CDCs mediate membrane binding in part through a conserved C-terminal undecapeptide, which contains a highly conserved and reactive cysteine residue. In CDC listeriolysin O, this Cys residue (Cys484) was shown to be post-translationally modified by GS-ylation [39]. It has been observed that the GS-ylation of Cys484 completely abolishes the activity of the toxin, and the inhibitory effect is fully reversible in the presence of GSH. It is possible that GS-ylation protects the cysteine residue from irreversible oxidation while its reduction is involved in carrying out the toxic activity of the protein. Therefore, GS-ylation may constitute a conserved post-translational regulatory mechanism necessary for the optimal activity of the toxin [39].

### 3.7. Synechocystis *sp.*

**Figure 2:**
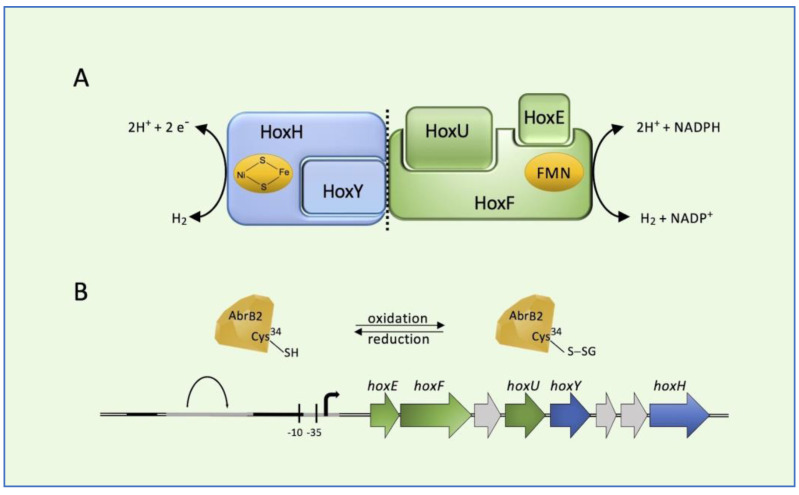
Bidirectional [NiFe]-hydrogenase (HoxEFUYH; Hox for ***h***ydrogen ***ox***idation) from cyanobacterium *Synechocystis* PCC 6803. (**A**) Schematic representation of the heteropentameric enzyme and (**B**) its corresponding genes in the *hox* locus. AbrB2 (*a*ntibiotic *r*esistance) regulator is represented as a diamond. Black bars: LexA (locus for X-ray sensitivity A) binding region; gray bars: AbrB-binding region.

Cyanobacteria is a phylum of the most diverse and widely distributed prokaryotes able to perform oxygenic photosynthesis [40]. Cyanobacterium *Synechocystis* PCC 6803 possesses a bidirectional [NiFe]-hydrogenase (HoxEFUYH; Hox for ***h***ydrogen ***ox***idation), usually coupling protons and electrons/hydrogen interconversion at the [NiFe] active site with NADPH as the electron donor (or vice versa) at the flavin mononucleotide active site [41]. In cyanobacteria, bidirectional hydrogenases are mainly involved in the removal of excess electrons derived from fermentation and photosynthesis, resulting in hydrogen production [42]. The heteropentameric enzyme is composed of the hydrogenase sub-complex (HoxYH) and the diaphorase sub-complex (HoxEFU) (Figure 2A). In addition to principally catalyzing reversible hydrogen oxidation, the [NiFe]-hydrogenase is involved in other processes. For example, under fermentative growth conditions, the production of hydrogen occurs as a means of maintaining redox poise. The *hoxEFUYH* operon is expressed under the positive control of LexA and AbrB1 regulators, and it is repressed by the negative regulation played by the binding of AbrB2 to its promoter [43,44]. It has been reported that the AbrB2 regulator can be post-translationally controlled through GS-ylation [45]. Indeed, a conserved cysteine residue at position 34 of AbrB2 is involved in the repressor activity (Figure 2B) and this cysteine is the target of GS-ylation, which regulates the binding of the *hox* promoter [45]. Under oxidative stress, AbrB2 is oxidized and Cys34 is glutathionylated. After recovery from oxidative stress, AbrB2 activity is retrieved by deglutathionylation. Thus, GS-ylation constitutes a significant tool for the regulation of cyanobacterial metabolism under oxidative stress conditions. This was also suggested by a large-scale proteomic analysis of GS-ylation in the cyanobacterium *Synechocistis* [46].

### 3.8. Glutathionylspermidine S-Thiolation

**Figure 3:**
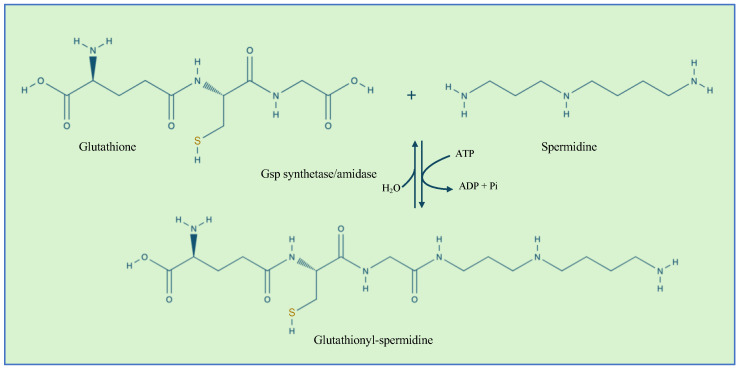
The reaction catalyzed by Gsp synthetase/amidase. The enzyme contains an N-terminal amidase domain and a C-terminal synthetase domain.

In Gram-negative bacteria, another defense mechanism against cysteine overoxidation is the formation of mixed disulfides between glutathionylspermidine (Gsp) and the reactive thiols of cysteine residues of proteins [47]. In addition, Gsp was found to display more reduced efficiency than GSH. Gsp synthesis is catalyzed by the Gsp synthetase/amidase through the reaction between the glycine carboxylate of the GSH and spermidine, a polyamine present in both eukaryotic and prokaryotic cells (Figure 3). Gsp synthetase/amidase is a bifunctional enzyme made of two domains that are able to catalyze the synthesis and the hydrolysis of Gsp, respectively (Figure 3). Protein S-thiolation with Gsp was first observed in *E. coli* [48]. It was also observed that the levels of Gsp S-thiolated proteins were increased by oxidative stress. In the presence of oxidative stress, Gsp amidase activity is inhibited by the oxidation of Cys59 residue. The inhibition of the enzyme activity leads to an increase in the concentration of Gsp followed by an accumulation of Gsp S-thiolate proteins [48]. Gsp S-thiolation has also been detected in *Salmonella* Typhimurium [49]. Table 1 summarizes the main target proteins undergoing GS-ylation.

## 4. Conclusions and Future Directions

The survey of the literature that we performed in the preparation of this short review revealed, on the one hand, that GS-ylation is a common mechanism to control protein function in bacteria belonging to different phyla but, on the other hand, still little is known on this PTM in prokaryotes when compared to eukaryotes. Given the vulnerability of proteins’ cysteine residues to conditions of oxidative stress, it is not surprising that one of the main roles of this modifier in bacteria, either synthesized by the microorganism itself or imported from the environment, is to provide protection from cysteine oxidation and consequent protein inactivation. In this regard, GSH is not the only resource used by bacteria, which can also in some cases rely on different thiols like mycothiol and bacillithiol or on the shorter version of GSH, i.e., gamma-glutamylcysteine, which is used by selected Gram-positive bacteria [18]. However, a number of studies surveyed here have also highlighted more specialized roles for GS-ylation in influencing important cellular events, ranging from the control of the DnaK interactome in *E. coli* to the control of phagocytosis suppression in *Y. pestis* and the escape from the phagolysosome of *L. monocytogenes*—events that underlie a considerable role of this PTM in virulence—to the control of gene transcription in cyanobacteria.

It is important to underline that many of these interesting studies that shed light on the specialized roles of GS-ylation in bacterial pathogenicity and interspecies competition and survival are quite recent and testify that this field of research is active and lively. Whole proteome PTM analysis of many different bacteria in various conditions, using mass spectrometry methods, are performed routinely today and hold the promise to identify new proteins and new mechanisms that are affected by GS-ylation in bacteria and enhance our understanding of the physiological and pathological roles played by this important PTM in bacteria.

GS-ylation in bacteria is a promising and under-explored field with many future directions, as a complete understanding of the functions of this PTM in bacteria has not yet been achieved. We have reviewed here several recent discoveries on the role of GS-ylation in oxidative stress response, virulence regulation, biofilm formation, and adaptation to environmental stress. These findings, and possibly others that will emerge from future research in this field, promise to allow for the identification of new therapeutic targets for the control of bacterial infections, especially in the face of increasing antibiotic resistance, and we believe that specific inhibitors or activators of GS-ylation pathways in different bacteria will be developed in the near future for this purpose.

## Figures and Tables

**Table 1:** GS-ylations and their functional role in bacteria.

Organism	Target Protein	Target Residue	Effect of GS-Ylation	Ref.
*Streptococcus mutants*	thioredoxin-like protein	Cys41	Protection from toxic effect of ROS	[22]
*Streptococcus pneumoniae*	NmlR	Cys52	Redox-sensing transcriptional activator of the *adhC* geme	[24]
*Escherichia coli*	DnaK	Cys15	Inactivation of DnaK	[30]
*Yersinia pestis*	LcrV protein	Cys273	Resistance to phagocytosis	[32]
*Acidithiobacillus caldus*	Persulfide dioxygenases	Cys87/Cys224	Protective mechanism against uncontrolled thiol oxidation	[37]
*Listeria monocytogenes*	listeriolysin O	Cys484	Inhibition of toxin activity	[39]
*Synechocystis* sp.	ABrB2 regulator	Cys34	Protection from oxidative stress	[45]

## Data Availability

No new data were created or analyzed in this study.
